# What have we learned from the time trend of mass shootings in the U.S.?

**DOI:** 10.1371/journal.pone.0204722

**Published:** 2018-10-18

**Authors:** Ping-I Lin, Lin Fei, Drew Barzman, M. Hossain

**Affiliations:** 1 Department of Health Sciences, Karlstad University, Universitetsgatan, Karlstad, Sweden; 2 Division of Biostatistics and Epidemiology, Cincinnati Children’s Hospital Medical Center, Cincinnati, OH, United States of America; 3 Division of Child and Adolescent Psychiatry, Cincinnati Children’s Hospital Medical Center, Cincinnati, OH, United States of America; University of California Irvine, UNITED STATES

## Abstract

Little is known regarding the time trend of mass shootings and associated risk factors. In the current study, we intended to explore the time trend and relevant risk factors for mass shootings in the U.S. We attempted to identify factors associated with incidence rates of mass shootings at the population level. We evaluated if state-level gun ownership rate, serious mental illness rate, poverty percentage, and gun law permissiveness could predict the state-level mass shooting rate, using the Bayesian zero-inflated Poisson regression model. We also tested if the nationwide incidence rate of mass shootings increased over the past three decades using the non-homogenous Poisson regression model. We further examined if the frequency of online media coverage and online search interest levels correlated with the interval between two consecutive incidents. The results suggest an increasing trend of mass shooting incidences over time (p < 0.001). However, none of the state-level variables could predict the mass shooting rate. Interestingly, we have found inverse correlations between the interval between consecutive shootings and the frequency of on-line related reports as well as on-line search interests, respectively (p < 0.001). Therefore, our findings suggest that online media might correlate with the increasing incidence rate of mass shootings. Future research is warranted to continue monitoring if the incidence rates of mass shootings change with any population-level factors in order to inform us of possible prevention strategies.

## Introduction

Although mass shootings are rare violent behaviors compared with other violent crimes, these incidents adversely impact the society as a whole. A better understanding of risk factors associated with such violent behaviors could provide an initial key to possible intervention strategies. There have been only a handful of published studies that identified risk factors for mass shootings. The limited published findings may be, at least in part, attributable to their rarity that strains the analytic techniques. To partially circumvent this technical challenge, one may explore risk factors at the population level, which may shed some light on “points of prevention” from the perspective of public health. Therefore, we attempted to identify several population-level risk factors associated with such incidents in the current study.

The literature suggests several risk factors associated with homicides which may provide some clues to possible risk factors for mass shootings. Accumulating evidence suggested that gun ownership is associated with gun-related deaths. Although firearm-related murder rates are related to area-based gun ownership rate [1–4], it remains unclear whether incidences of mass shootings are attributable to the gun ownership rate. Emergent, albeit inconsistent, evidence has also indicated that mental health may play a role in mass murders [5, 6]. Poverty has been documented to be associated with the homicide rate, but, to our knowledge [7–11], its link with mass shootings has not be evaluated yet. Another population-level risk factor associated with mass murders is the “contagion effect,” which refers to the phenomenon a murder may temporarily increase the probability of a similar event in the proximal future [12, 13]. It remains unclear if such a contagious effect is triggered by increased attention, which may be reflected by the quantity of media coverage of the key event in the community. Media exposure of mass violent incidents has been found to adversely impact mental health of children and adolescents [14]. A Finnish study reports that media exposure was positively associated with the post-traumatic stress symptoms following a school-shooting event [15]. Research prior to 1990 has shown that behaviors and value systems are shaped by media to some degree [16]. However, the link between media exposure to violent behaviors in children and adolescents remains controversial [17]. Heterogeneous research methods may have caused the inconsistent findings. There has been scarce published research about the impact of media exposure on mass shooting incidents. Furthermore, little is known about whether media coverage on such incidents may contribute to the time trend of such extreme violence.

The limited understanding of mass shootings may have hindered the progress in dealing with this rising threat to the public health. We proposed to address three major questions in this study as follows. (1) *What are the population-level factors associated with the probability of mass shootings*? (2) *Is the incidence rate of mass shooting increasing during the past three decades*? (3) *Is the online media associated with the probable “contagious effect*?” We believe that the answers to these three major questions will clarify some population-level risk factors for mass shootings, and provide some clues to how the public policies should react to this problem if these risk factors are modifiable (e.g., poverty rate). In the current study, we have gathered relevant information about mass shootings that occurred within the U.S. in the past 30 years to assess whether there are any systematic temporal and spatial patterns and to examine whether these incidences can be explained by risk factors.

## Methods

### 1. Data source

The definition of such an event is critical in the research on mass shootings. The definition of “mass shooting” is particularly critical in the current study since the rates of such an event is the key outcome. In our analyses, we defined a mass shooting as an act of firearm violence that resulted in at least four fatalities (not including the perpetrator), at the same time, or over a relatively short period of time in the case of shooting sprees. This definition is partially based on the definition of “mass murder” from a report of FBI’s Behavioral Analysis Unit [18]. Additionally, we attempted to focus on the mass shootings unlike conventional homicidal behaviors. As stated in “A Guide to Mass Shootings in America,” the editors of Mother Jones’ Website wrote, “*Our research has focused on indiscriminate rampages in public places resulting in four or more victims killed by the attacker*. *We exclude shootings stemming from more conventional crimes such as armed robbery or gang violence*.”We, therefore, chose Mother Jones’ website as the main source of our data, where 100 mass shootings were selected from January of 1982 to May of 2018 [19]. The data related to the state-level gun ownership rate were estimated from the proportion of firearm-related suicides in all suicides acquired from the WISQARS database associated with the Center for Disease Control (CDC) [2]. We first selected “suicide” in the section entitled “Intent or manner of the injury,” and then selected “Firearm” versus”Non-Firearm" in the section entitled “Cause or mechanism of the injury" to extract the state-specific numbers of firearm-related suicides and non-firearm-related suicides from 2013 through 2016, respectively. The gun ownership was then calculated as the average number of firearm-related suicides divided by the average number of all suicides during this period of time. The data of state-level serious mental illness rates were extracted from the CDC Report: Mental Illness Surveillance Among U.S. Adults, which included the state-specific rate of “serious psychological distress” among adults aged ≥ 18 years [20]. Briefly, we used the online tool “Behavioral Risk Factor Surveillance System” to select the lifetime depression prevalence rate by state as a proxy for the state-specific mental illness rate. We further retrieved the data of poverty rates by state from the United States Census Bureau by selecting the “Income and poverty" table by each state during 2013 through 2016 available on QuickFacts webpage [21].

The number of on-line reports about mass shootings were extracted using the Google searching engine. We used the “allintex” function in the Google search engine, and then specified the time frame using the function of “research tools” to calculate the number of on-line posted articles between two consecutive incidents. Briefly, we have used the following keywords: “mass shooting/shootings” or “rampage shooting/shootings” to extract all on-line public reports and posts that discussed mass shooting following a specific mass shooting event. The media coverage density (i.e., daily number of online reports and posts) was then calculated as the number of on-line articles divided by the length of the period between the relevant incident and the next incident. Previous studies have shown that the Google searching engine can effectively retrieve the information about the impact of the media [22, 23]. We also compiled the data of “internet search interest,” which was derived from the *Google Trends* database. We extracted the data related to interest levels of internet searching using the key word, “mass shooting” or “rampage shooting” in the U.S. during 2005–2017. Search interest was defined as the relative percentage of searches compared to the peak in the specified time period. We chose to focus on the data after January 1, 2004 since Google Trends started to store the data of users’ internet search histories in late 2004. The detailed raw data are stored in a cross-disciplinary repository, figshare [24,25].

### 2. Statistical methods

To investigate the impact of state-level gun ownership rate on the mass shooting rate, we built a Poisson model for state-specific incidence count with other covariate effects such as state specific serious mental illness rate and poverty percentage, and population size as an offset variable in a Bayesian framework, with two random effects for structured and unstructured spatial correlation structure. Since there were no mass shooting incidents in 19 states during this period of 32 years, we used zero-inflated Poisson count model to adjust for the excessive zeroes. All incidents were summarized by state as counts, regressed on gun ownership rate through a log-link function as,

$$\log(\mathrm{mean}\;\mathrm{count}\;\mathrm{for}\;\mathrm{state}\;i)={log(\mathrm{Population}\;i)+\beta }_{1}+\beta_{2}∙(\mathrm{Gun}\;\mathrm{Ownership}\;\mathrm{Rate}\;i)+\beta_{3}∙(\mathrm{Serious}\;\mathrm{Mental}\;\mathrm{Illness}\;\mathrm{Rate}\;i)+\beta_{4}∙(\mathrm{Poverty}\;\mathrm{Percentage}\;i)+v_{i}+u_{i}$$ (1)

where *v*_*i*_ is structured random state effect with an intrinsic conditional autoregressive (CAR) model was specified as a prior distribution, and *u*_*i*_ is unstructured random effect with an exchangeable normal distribution with zero mean as a prior distribution. The use of spatial model, specifically the CAR model was intended to adjust for the effect for similar neighborhood characteristics among the states sharing common borders. This model can examine not only the state’s mass shooting rate but also the corresponding rates in all surrounding states. Therefore, spatial modeling could enhance both the estimates and our knowledge of the degree of uncertainty associated with these estimates, and may overcome the effect of artificial state boundaries. The model for the excess zeroes considers the same predictors as the main Poisson model. In addition, we also treated the logarithms of state-specific population size as an offset, instead of a covariate, to examine the relationship between covariates and probability of shootings.

To evaluate if the incidence rate was increasing during the past three decades, we implemented a non-homogenous Poisson regression model on biannual incidence count for the U.S. with a linear *year* predictor to characterize the time trend. A quadratic term turns out to be not significant. Formally, the Poisson regression model is,

$$\mathrm{Nationwide}\;\mathrm{count}\;\mathrm{from}\;2‑\mathrm{year}\;\mathrm{time}\;\mathrm{period}\;i=\beta_{1}+\beta_{2}∙(\mathrm{years}\;\mathrm{since}\;1982)$$ (2)

We also analyzed the annual count of mass shootings using a similar model for comparison of effects and model’s goodness of fit based on Akaike information criterion (AIC). We also assessed if inter-incidence time intervals (in days) decreased in recent years. Assuming the time intervals follows a time-dependent exponential distribution, we would be able to predict the risk of future incidences based on the incidence rate in the preceding two-year period.

We further investigated the speculated relationship between mass shootings and two online media indexes: (1) online media coverage and (2) online search interest. We hypothesize that the higher online media coverage density (i.e., the frequency of online media attention to the theme of “mass shootings” in a certain period of time) after a particular shooting might be associated with the shorter interval between this incident and the next one. We, therefore, calculated the online media coverage density by calculating the daily number of reports and posts about mass shootings during the interval between two consecutive incidents to investigate if media attention between an earlier shooting event could correlate with “when” the next shooting might occur. To alleviate the confounding effect due to the time trend that Google became the dominating Internet searching service provider in the late 2004, we only analyzed a subset of mass shooting incidents in our data set that occurred between January of 2005 and May of 2018. The distributions of media coverage density were highly skewed. Hence, we log-transformed the variable of media coverage density before we performed the Poisson regression model to see if the between-incident interval could be predicted by media coverage density adjusting for number of fatalities and the number of injuries, with time as an offset. Similarly, we tested if the same outcome could be predicted by the level of Internet search interest. Finally, we evaluated the associations between the outcome and both online media coverage density and search interest levels by adjusting the numbers of fatalities and injuries in the same Poisson model.

## Results

**Fig 1:**
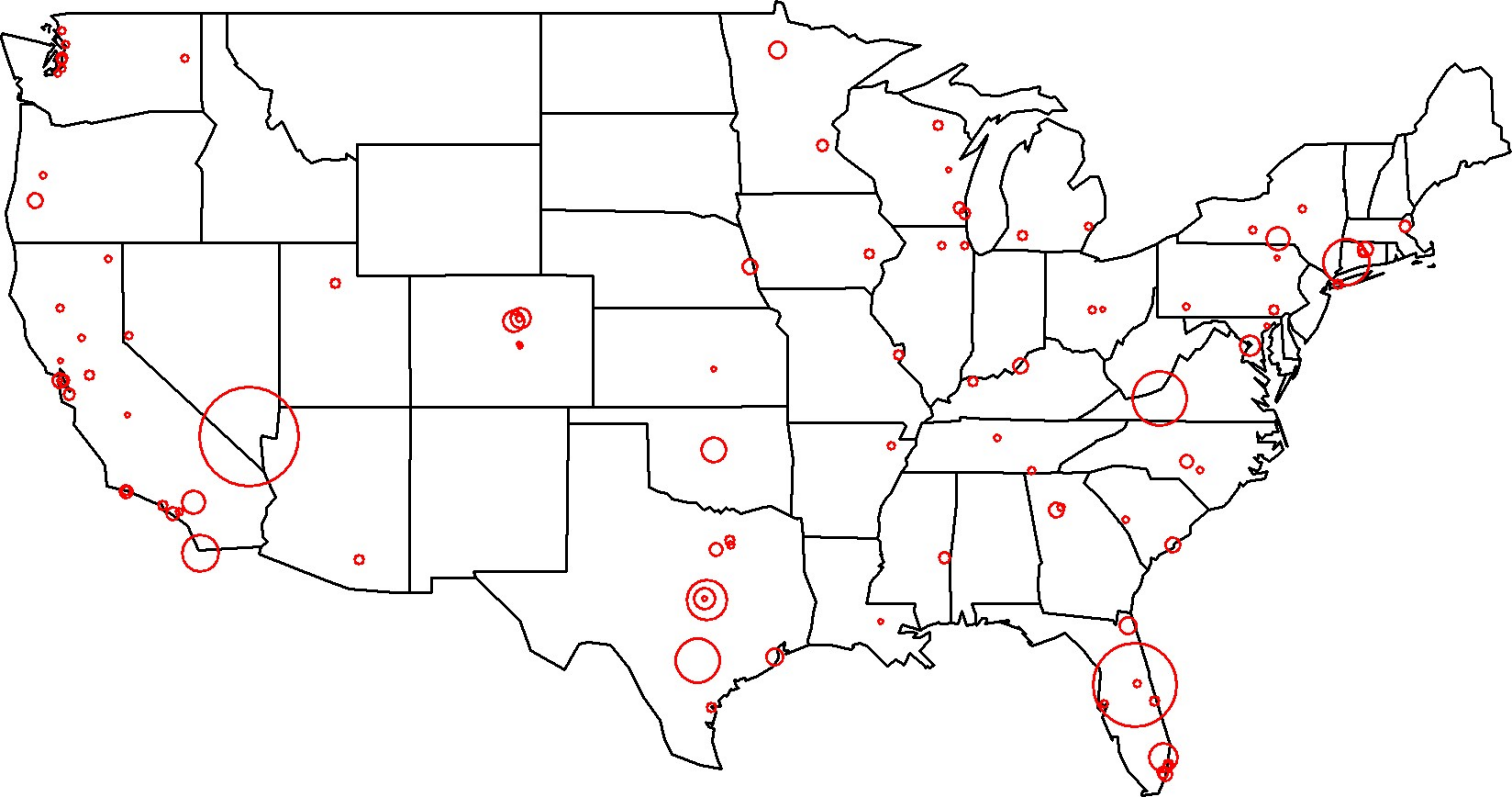
Geographic locations of shootings and respective fatalities (proportional to circle diameter) are presented.

There was a total of 100 mass shootings in the U.S. recorded in our compilation of data from January 1982 to May 2018, with 833 fatalities, and 1,292 injuries. The spatial locations of the shootings in the 48 states are displayed in Fig 1. Table 1 displays the results from the fitted Bayesian zero-inflated Poisson regression model which show that no state-level variables were statistically significantly associated with the number of mass shootings in each state. The goodness of fit value based on Deviance Information Criterion (a.k.a., DIC) of the zero-inflated Poisson regression model was 102.5 compared with the Poisson regression model, of which the DIC value was 145.7. Therefore, the Bayesian zero-inflated Poisson regression model still had a better model fit than the Bayesian Poisson regression model.

**Table 1:** Bayesian analysis estimates for the zero-inflated Poisson regression model with population size as an offset are shown.

Parameter	Mean	SD	2.5% Percentile	Median	97.5% Percentile
Intercept	-1.23	2.99	-6.9	-1.49	5.06
FS/S*	-0.35	1.24	-2.82	-0.35	2.19
Serious mental disorder rate	0.02	0.15	-0.29	0.04	0.29
Poverty rate	-0.02	0.05	-0.07	0.018	0.13
Gun law permissiveness	-0.26	0.39	-1.06	-0.25	0.49

* FS/S denotes the ratio of firearm-related suicides divided by all suicides.

**Fig 2:**
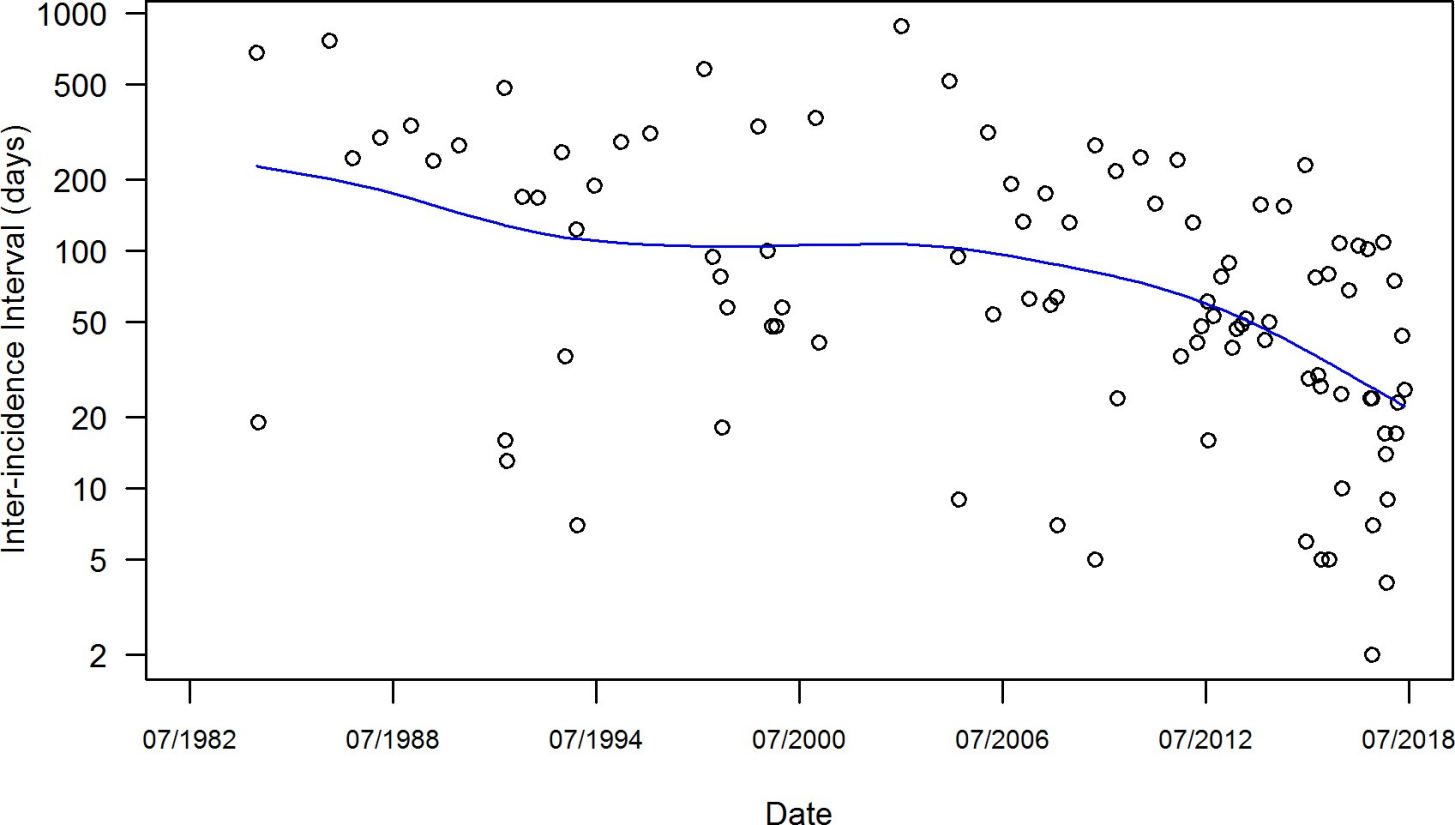
Interval time between mass shootings and its GAM fit for trend is shown.

The two Poisson regression models that examined the incidence rate show that the frequency of mass shootings has been increasing in recent three decades (p-value < 0.001). According to the AIC level, the model that focused on the biannual incidence rate may have a better goodness of fit than the model that focused on annual incidence rate (AIC levels were 93 and 122, respectively). The biannual incidence rates are shown in the S1 Fig. The frequency can be reflected by the time interval between two consecutive incidents, also known as inter-arrival time in a Poisson process. We plotted inter-arrival times (Fig 2) using a GAM fit (generalized additive model with smoothing spline of 4 degrees of freedom), to illustrate the time trend of incidence rates. If the trend is not present, i.e. the Poisson process is homogeneous, the inter-arrival time would follow a fixed exponential distribution with an intensity parameter *λ*. We estimated this parameter by a maximum likelihood estimate, and converted it to a monthly rate, ${\hat{\lambda }}_{M}=0.227$. This value could inform us of the risk for future mass shootings in the U.S.

To model the temporal trend for incidence rates, we adjusted the model with a time-varying Poisson process, i.e. a non-constant rate *λ* for the exponential distributions of inter-arrival time. In this model, the counts from every 2 years were calculated. The log(*λ*(*t*)), the logarithm of two-year expected count, was treated as a linear function of time. The resulting regression model was as follows:

log(λ(t))=0.55683+0.05033×(Year−1982)

The regression coefficient was highly significant (p < 0.001), but the quadratic term in the model was not significant. The fitted regression line super-imposed on the distribution is shown in S2 Fig.

According to the Poisson model, the possible over-dispersion parameter was estimated to be 1.53, which indicates a moderate effect. If the current frequency trend continues, we can assess the risk by adopting the monthly intensity rate from the above non-homogeneous Poisson model for the most recent year, ${\hat{\lambda }}_{M}=0.468$ (see the 3^rd^ column of Table 2).

**Table 2:** Predicted probability of at least one mass shooting within next few months.

Within months (*t*)	Probability of a shooting 1 − *e*^−0.227*t*^ Using constant rate	Probability of a shooting 1 − *e*^−0.468*t*^ Using regression model (2) predicted most recent yearly rate
1	0.203	0.374
2	0.366	0.608
3	0.494	0.755
6	0.745	0.940
9	0.871	0.985
12	0.935	0.996

**Fig 3:**
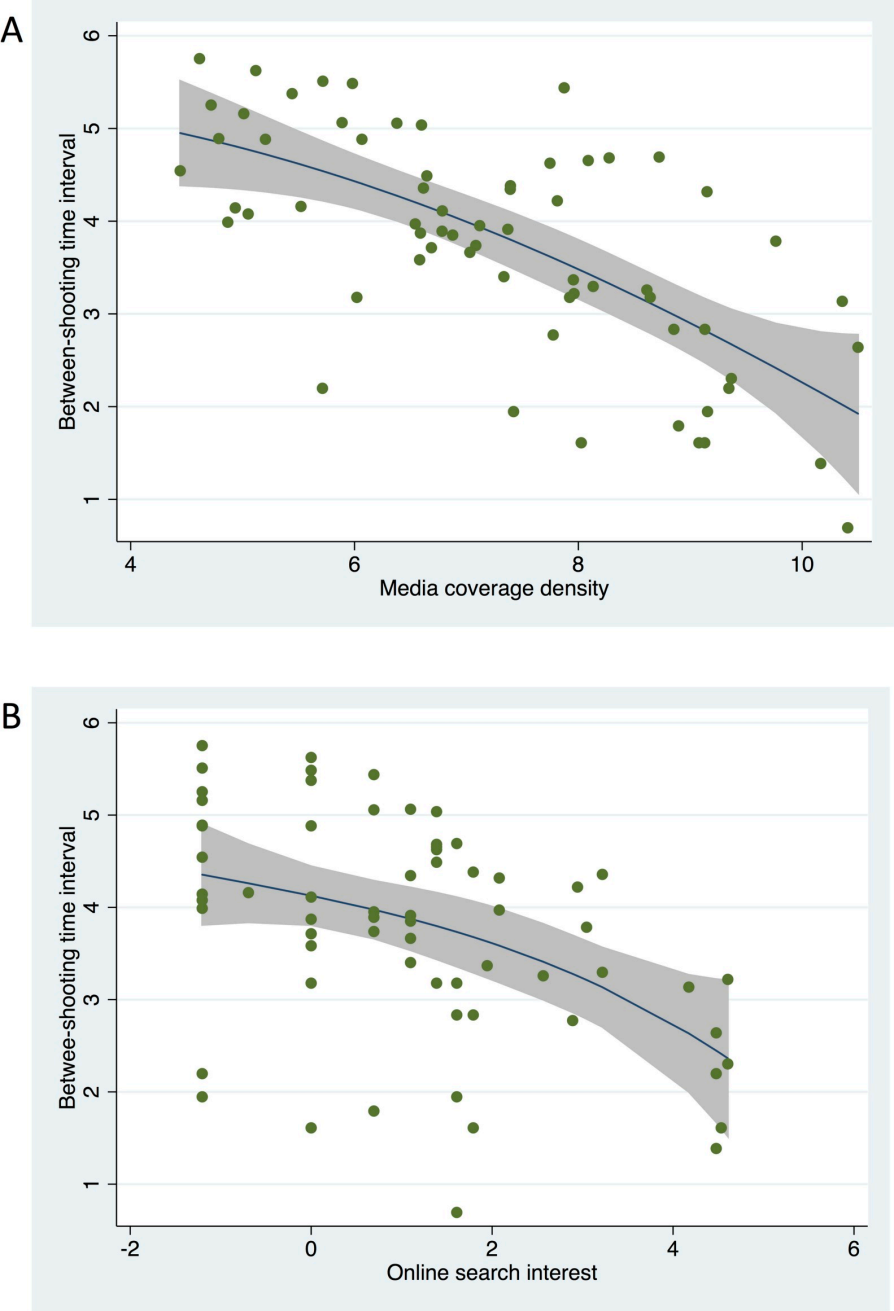
The relationship between online media coverage density (i.e., number of public posts and reports per day (panel A) and online search interest (panel B) for the preceding shooting incident and the interval between two consecutive shooting incidents is illustrated. The trend is presented as a polynomial smoothed line with 95% CI superimposed on the scattered plot.

In an attempt to explain this time trend, we performed a pair-wise correlation analysis of online media coverage, online search interest levels, and mass shootings. The correlation analysis results are summarized in S1 Table. It shows that both media coverage and search interest were inversely associated with the interval (Spearman’s correlation coefficient *ρ* = -0.70, p < 0.001). Fig 3 shows the local polynomial smoothed line that indicates the relationship between the on-line media indexes (i.e., number of public reports and posts per day and search interest, respectively) and the interval between two consecutive incidents. The two indexes were both inversely correlated with the time interval between two consecutive incidents. We further found that the temporal order (the calendar time since 1982 in our case) was also inversely associated with the interval (*ρ* = -0.37, p = 0.001). Because of the significant correlation between media coverage and temporal order, we used a ridge regression of inter-arrival interval on media coverage and time. Media coverage remained significantly correlated with between-incident interval after controlling for temporal order based on the ridge regression model (coefficient = -0.147, p = 0.006). Since internet search level was highly correlated with media coverage, the final fitted model selected media coverage over internet search interest levels.

## Discussion

Our study has attempted to answer three important questions concerning population-level risk factors associated with mass shootings, increasing rate of mass shooting, and the contagious effect. The results suggest that the neither proportion of suicides by firearms (as a proxy for gun ownership), poverty rate, nor serious mental illness rate, could significantly predict the state-specific mass shooting rate. However, the both annual and bi-annual incidence rates of mass shootings in the U.S. have been found to steadily increased during the past 30 years. We further observed that increasing online media attention is correlated with decreasing intervals between shooting incidents–which might be considered to lend some support to the hypothesis of a “contagious effect”.

Both gun ownership and the rate of mass shootings declined simultaneously in Australia during 1979–2013 [26]. Our study did not obtain similar findings related to gun ownership, which might be partly attributable to our use of a different measurement to calculate the proxy for “gun ownership” due to the limited data on gun ownership in the U.S. Notably, the Australia study had a stricter definition of mass shooting (i.e., at least five victims that were murdered). The relationship between the gun ownership and mass shooting rate might be confounded by gun law permissiveness, since gun law permissiveness was found to influence the homicide rate [27]. We hence propose an alternative hypothesize that “gun law permissiveness” may influence either the gun ownership or shooting rate so it could be considered as a confounder. There are only nine states where law enforcement officials could exert wide discretion over whether to issue concealed-carry permits. These states are California, Connecticut, Delaware, Hawaii, Maryland, Massachusetts, New Jersey, New York, and Rhode Island. This statement was based on individual state laws, which include California Penal Code § 26150, § 26155, Connecticut General Statutes § 29–28, Delaware Code § 1441, Hawaii Revised Statutes § 134–9, Maryland Public Safety Code § 5–306, Massachusetts General Laws 140 § 131, New Jersey Statutes § 2C:58–4, and General Laws of Rhode Island § 11-47-11. The results, based on Bayesian zero-inflated Poisson regression model show that gun law permissiveness was only nominally correlated with gun ownership (Kendall correlation test p-value = 0.06), and gun ownership was not statistically associated with the mass shooting rate with or without gun law permissiveness being adjusted in the statistical model (Table 1).

Our data suggest that the state-level rate of serious mental illness could not predict the mass shooting rate. There is evidence for an increased prevalence and severity of mental illness in adults in recent years [28, 29]. For example, from 1991–1992 to 2001–2002, the U.S. adult prevalence of major depression significantly increased from 3.33% to 7.06% [29]. However, such studies are difficult to interpret because the increased prevalence may be related to increased help seeking behavior, better screening, or other confounding factors [28]. The link between mental disorders and mass shootings may be one of the problematic self-evident assumptions which tend to oversimplify the causes of such a violent behavior [30]. Future research is needed to investigate any correlations between the increased frequency of mass shootings, types of mental illness, psychiatric medication non-adherence, and increased need for more psychiatric services.

Our results suggest that the on-line media coverage of the current shootings as well as internet search interest levels may predict how soon the next shooting tragedy may occur. The positive correlation between on-line media coverage and search interest might provide some clues to the predisposing factors underlying the contagion effect. However, our estimates based on the population-level data may not reflect the genuine influence of media exposure on individuals. With the growing popularity of social networking on-line media, the media exposure to mass shootings solely based on the internet-based reports may underestimate the total level of relevant media exposure since most of the social networking media-related activities cannot be directly accessed through on-line data queries. Although we cannot rule out some confounders for the relationship between media coverage and the rising incidence of mass shootings, media exposure seems to play a role. There have been several recent studies using Google to study social behaviors–three have been focused on violent behaviors [31–34]. Therefore, the high-efficiency Internet search engine has the reliable capacity of capturing the “interest level” associated with violence in at least Internet users.

Our media-related analysis has several limitations. First, we only focused on the most proximal preceding incident, and hence we ignored the impact of media coverage on more distant incidents. Although the media coverage on cumulative mass shooting incidents is overall greater at more recent time-points, compared to earlier incidents, we could not rule out the confounding effect of some incidents that generated more media attention despite being temporally more distant. This confounding effect, if present, would by default make more recent incidents subject to higher levels of accumulative influence of media. We have adjusted for the temporal order and found the effect of media coverage remained significant. Meanwhile, we also examined the role of on-line search interest levels in the incidence of mass shootings. On-line search interest did positively correlate with on-line media coverage. Therefore, we might infer that the frequency of on-line related reports might reflect both interest levels of the media providers and consumers, and vice versa. Second, we calculated the media coverage by counting the number of on-line reports. However, the emergence of social networking web resources may play a role in the time-dependent increment of media exposure to these incidents; such a confounding effect of social networking could not be measured with the current analytic methods. Similarly, the internet search interest levels could not capture the interest levels of consumers in the social media. Although on-line news reports and articles only represent a fraction of media exposure related to mass shootings, on-line articles, especially those related to social networking (e.g., Twitter), may correlate with other types of media coverage, such as television [35]. Therefore, on-line news reports might serve as a proxy for media exposure to a particular mass shooting incident. Meanwhile, the frequency of internet searches for relevant key words may indicate interest levels for how consumers react to the on-line media. Additionally, although our data of media coverage density in each time interval between two consecutive mass shooting incidents was supposedly primarily derived from the reports in response to mass shootings that met the Mother Jones’ narrow criteria (i.e., one perpetrator and at least 4 victims), some reports might actually discuss other mass shooting events with lower levels of fatalities (i.e., broad definition of mass shootings). Such a “mismatch” due to the mix of media attention towards both narrow-definition mass shootings and broad-definition mass shootings might be jointly considered as media attention associated with the time interval between two narrow-definition mass shootings. Therefore, our data actually suggest that increasing media attention towards various types of mass shootings jointly correlate with increasing incidence rate of mass shootings.

Recent evidence suggests a “contagion” in mass shootings–which refers to the phenomenon that a mass shooting may temporarily increase the probability of a similar event in the immediate future [12]. The “contagion” phenomenon has been well documented in other forms of violent behaviors, including suicides and homicides. The relationship between media and violent behaviors has been documented by several prior studies. For example, Cantora and colleagues have reported a series of mass-homicides occurring in Australia, New Zealand and the United Kingdom 1987–1996 in the context of possible media influences [36]. Similarly, Phillips and colleagues have raised the concerns that homicides seemed to have been “rewarded” by media attention [37]. Kostinsky and colleagues reported the clustering of threats of school violence following the Columbine massacre was initiated by imitation [38]. Although an earlier study did not find the clustering of rampage murders when they examined the data from 1988–1999 [39], recent evidence did suggest this phenomenon when more data were extracted [12, 13]. Taken together, these lines of evidence have shown the influence of media on clustering (or contagion) of mass shootings. However, caution needs to be exercised when we attempt to infer the “impact” of media coverage–in other words, we might at best infer that greater media coverage might predict an earlier onset of the next shooting incident, but we could not clarify whether the increased incidents could be attributable to increased media coverage.

To further examine if some specific shootings that receive unusually high media attention are outliers that bias the observation, we have also examined the post-event effects for three specific incidents: the mass shooting at Sandy Hook Elementary School in December of 2012, the mass shooting at Las Vegard strip October of 2017, and the mass shooting at Marjory Stoneman Douglas High School shooting in February of 2018. The first case triggered the presidential initiative of firearm regulation reform proposals; the second case caused the largest number of fatalities associated with mass shooting in the history of the U.S.A.; the third case triggered the largest nationwide protest that called for firearm regulation reforms. We found that the media index values, such as number of daily online articles and the online search interest level, were increased variably (Sandy Hook Elementary School shooting 3%, Las Vegard strip shooting 14%, and Marjory Stoneman Douglas High School shooting 241%). The second case caused increased media attention, and the interval between this case and its following shooting incident was 17 days, which was 0.7 standard deviation below the mean of the distribution of the log-transformed intervals. The third case apparently caused unusually heightened media attention, and the interval between the third case and its following shooting incident was 23 days, which was 0.5 standard deviation below the mean of the distribution of the log-transformed intervals. Therefore, these particular observations seemed to follow the trend that media attention was inversely correlated with the interval between the current shooting event and the next shooting event, especially for the more recent cases. Furthermore, we removed these “outliers” and re-ran the analysis. The results still show that higher internet media exposure was significantly correlated with a shorter interval between the corresponding shooting and the following shooting. Therefore, the original observations were not biased by the “outliers” that triggered unusually high media attention levels.

Another limitation lies in the assumptions in the process of statistical modeling. For example, we relied on the cross-sectional data to evaluate ownership rates, wherein a spatial correlation structure is needed with priors specified from a Bayesian analysis. Our statistical approaches were hence different from Wallace’s methods in a recent study, and hence we did not replicate the prior findings that gun ownership could account for the incidence of mass shootings [40]. Furthermore, we assumed shooting events were independent of each other in the Poisson regression model, while a linear time trend could exist. This concern might have been addressed by the validation of a significant effect of a second-order term. Finally, our assessment of future mass shooting risk was based on a narrow definition which might result in conservative estimates of the incidence rate and lead to biased effect sizes of risk factors. Similarly, inconsistent findings from the literature may, at least in part, arise from different definitions or classifications of predictors. Future research is needed to explore methodological challenges arising from the aforementioned issues in order to properly evaluate the time trend of mass shootings.

## Supporting information

S1 Table The Spearman correlation matrix for covariates associated with between-incident time interval is shown. (DOCX)

S1 Fig Bi-annual mass shooting frequencies, with a linear trend fit by a Poisson regression model, is illustrated. (TIFF)

S2 Fig Histogram of time between consecutive mass shootings (in days), along with its exponential fit, from February of 1982 through May of 2018, assuming a time-homogenous Poisson process, is shown. (TIFF)
