# Supplementary material for: What have we learned from the time trend of mass shootings in the U.S.?
Source: PLoS One. 2018 Oct 18;13(10):e0204722. doi: 10.1371/journal.pone.0204722 (PMC6193640; doi:10.1371/journal.pone.0204722)
Supplement: S1 Table — (DOCX) [file pone.0204722.s001.docx]

S1 Table. The Spearman correlation matrix for covariates associated with between-incident time interval is shown.

|  | Interval | Media coverage density | Search interest level | Fatalities | Injuries | Time order |
| --- | --- | --- | --- | --- | --- | --- |
| Interval |  |  |  |  |  |  |
| Media coverage density | -0.66** |  |  |  |  |  |
| Search interest level | -0.46** | 0.81** |  |  |  |  |
| Fatalities | 0.17 | -0.32** | -0.28* |  |  |  |
| Injuries | -0.05 | 0.05 | 0.02 | 0.46** |  |  |
| Time order^†^ | 0.43** | -0.91** | -0.85** | 0.36** | 0.03 |  |

* 0.01 ≤ p-value < 0.05

** p-value < 0.01

^†^ Time order: we ranked all cases by the temporal order, and assigned “1” to the most recent case in 2018 while “100” to the earliest case in 1982 (there are a total of 100 cases during the past three decades). Only cases after January 2005 were analyzed.
