# Supplementary figures and images for: What have we learned from the time trend of mass shootings in the U.S.?
Source: PLoS One. 2018 Oct 18;13(10):e0204722. doi: 10.1371/journal.pone.0204722 (PMC6193640; doi:10.1371/journal.pone.0204722)

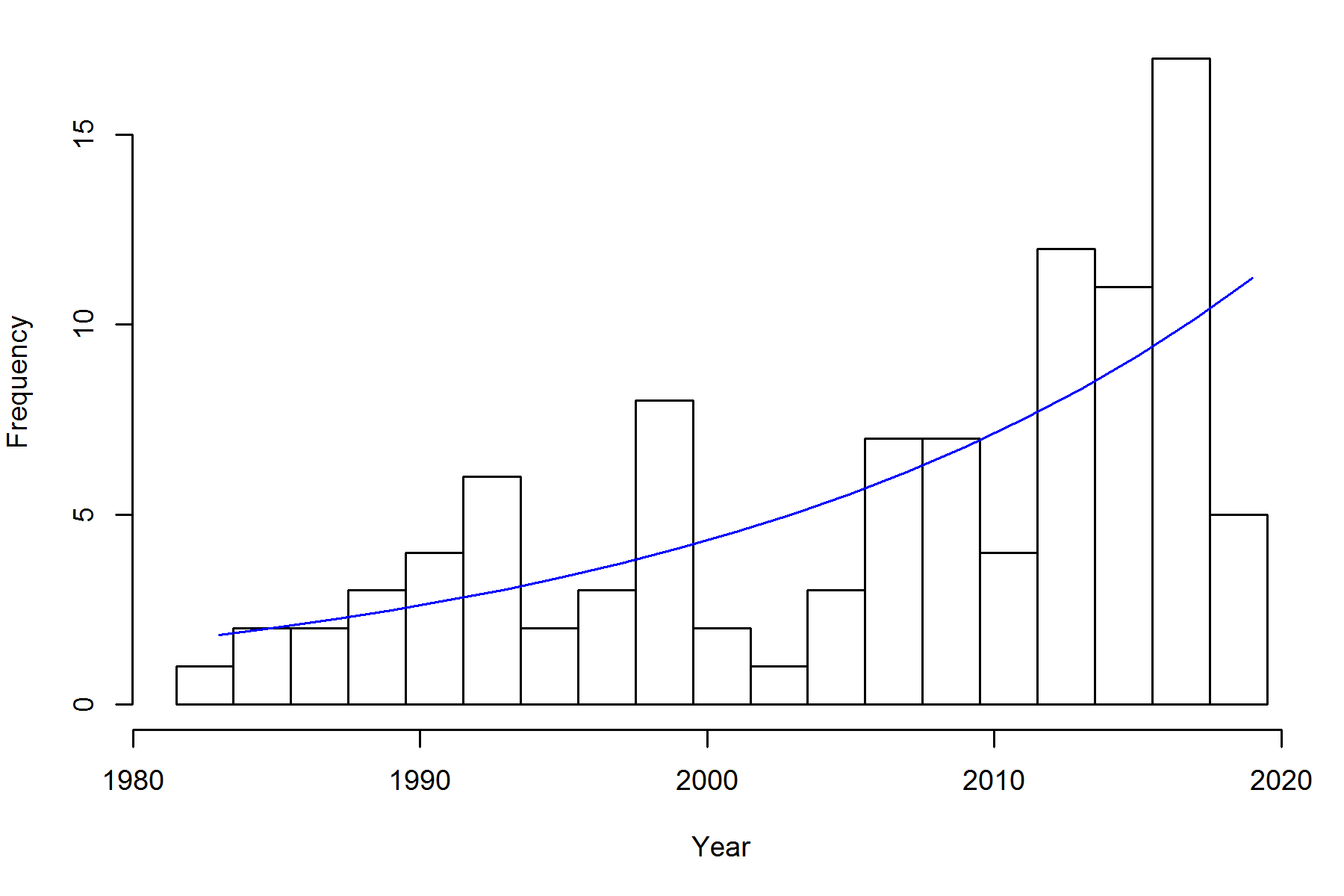

Supplement: S1 Fig — (TIFF) [file pone.0204722.s002.tiff]

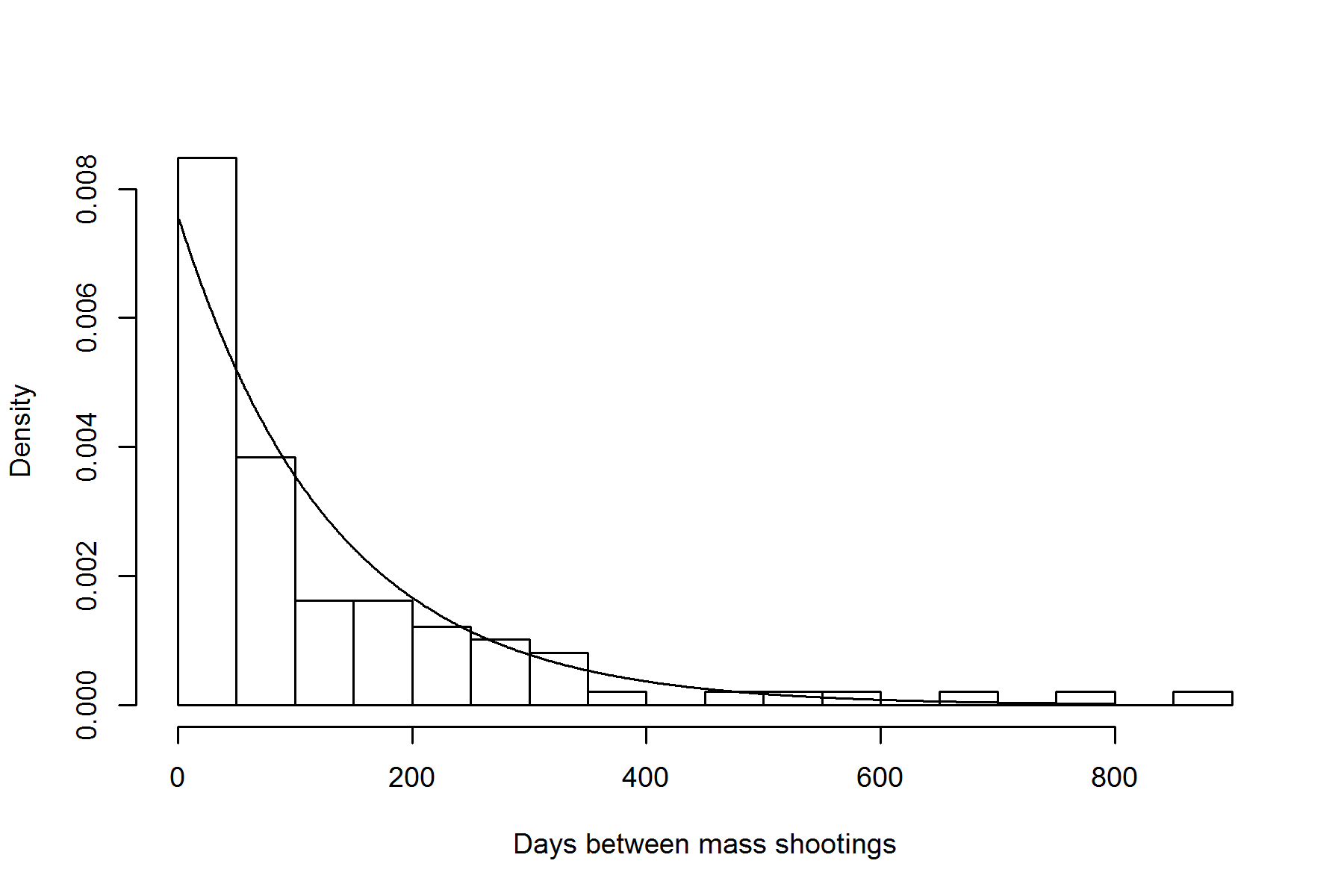

Supplement: S2 Fig — (TIFF) [file pone.0204722.s003.tiff]
